# An Efficient Building Extraction Method from High Spatial Resolution Remote Sensing Images Based on Improved Mask R-CNN

**DOI:** 10.3390/s20051465

**Published:** 2020-03-06

**Authors:** Lili Zhang, Jisen Wu, Yu Fan, Hongmin Gao, Yehong Shao

**Affiliations:** 1 College of Computer and Information Engineering, Hohai University, Nanjing 211100, China; 171307030009@hhu.edu.cn (J.W.); fanyu@hhu.edu.cn (Y.F.); gaohongmin@hhu.edu.cn (H.G.); 2 Arts and Science, Ohio University Southern, Ironton, OH 45638, USA; yehongshao@gmail.com

**Keywords:** building extraction, convolutional neural networks, mask R-CNN, high-resolution remote sensing image

## Abstract

In this paper, we consider building extraction from high spatial resolution remote sensing images. At present, most building extraction methods are based on artificial features. However, the diversity and complexity of buildings mean that building extraction methods still face great challenges, so methods based on deep learning have recently been proposed. In this paper, a building extraction framework based on a convolution neural network and edge detection algorithm is proposed. The method is called Mask R-CNN Fusion Sobel. Because of the outstanding achievement of Mask R-CNN in the field of image segmentation, this paper improves it and then applies it in remote sensing image building extraction. Our method consists of three parts. First, the convolutional neural network is used for rough location and pixel level classification, and the problem of false and missed extraction is solved by automatically discovering semantic features. Second, Sobel edge detection algorithm is used to segment building edges accurately so as to solve the problem of edge extraction and the integrity of the object of deep convolutional neural networks in semantic segmentation. Third, buildings are extracted by the fusion algorithm. We utilize the proposed framework to extract the building in high-resolution remote sensing images from Chinese satellite GF-2, and the experiments show that the average value of IOU (intersection over union) of the proposed method was 88.7% and the average value of Kappa was 87.8%, respectively. Therefore, our method can be applied to the recognition and segmentation of complex buildings and is superior to the classical method in accuracy.

## 1. Introduction

With the development of remote sensing satellite technology and the demand of urbanization, it has become an important research field to automatically and accurately extract building objects from remote sensing images. There are many studies on building extraction approaches from remote sensing images based on artificial design features, and these approaches can be divided into three categories. The first is the building extraction method based on edge and corner detection and matching. In this method, feature matching is carried out through edge and corner information of the building to complete building extraction [1]. The second is the building extraction method combined with elevation information, and this kind of method uses elevation information to separate out non-ground points, and then detect buildings by combining the common edge features, spectral features and other artificial features [2]. The last is the object-oriented building extraction method. This kind of method uses edge information to segment the remote sensing image initially so that homogeneous pixels make up objects of different sizes, then extract them by using the unique spectral information, shape and texture features of the buildings [3]. Due to different shooting angles, light and other factors, remote sensing images in different periods have considerable internal variability, and buildings have a variety of structure, texture and spectral information, therefore, the methods above cannot perform well in the extraction of complex buildings.

**Figure 1:**
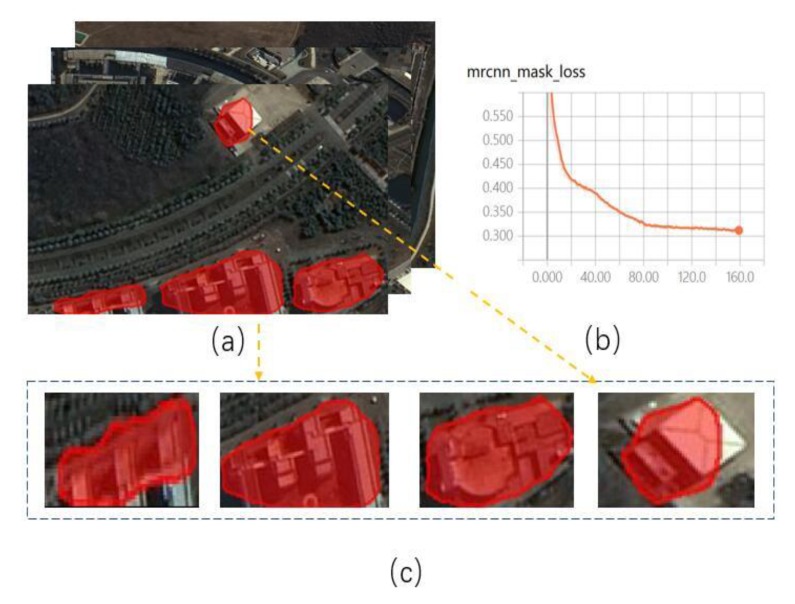
Building extraction based on Mask R-CNN. (**a**) The whole building extraction; (**b**) Loss function curve; (**c**) The single building extraction.

In recent years, deep learning technology has ushered in a new wave of revival. At present, deep learning technology represented by deep convolutional neural networks has achieved excellent results in the field of computer vision [4,5,6]. Compared with the traditional method of feature extraction in artificial design, deep convolutional neural networks can obtain the structure, texture, semantics and other information of the object through multiple convolutional layers, and their performance is closer to visual interpretation in object recognition. The experiments in [7,8] had the advantages of deep convolutional neural networks in the field of object detection, but also revealed the problems of local absence and edge blur in image segmentation. This phenomenon is especially serious when the hardware equipment is insufficient or the dataset is small. Figure 1 shows this phenomenon using a mask image [9]. The main reasons for poor extraction results are as follows. Firstly, the lack of data sets makes the convolutional neural network unable to effectively learn the hierarchical contextual image features. Secondly, adding more layers to the suitably deep model leads to higher training errors [10]. Lastly, the prediction ability of CNNs (convolutional neural networks) with low computation is at the expense of a decrease in output resolution.

This paper investigates the possibility of artificial features to optimize the building extraction results of convolutional neural networks. A fusion method of artificial edge features and convolutional neural network recognition results is proposed to address the problem of building extraction accurately.

## 2. Related Work

(A) Artificial design model: this model based on artificial features has been widely used in the detection of remote sensing images. Combining image segmentation technology, spectral constraint, shadow constraint and shape constraint, Ding et al. proposed a new building extraction framework based on the MBI (morphological building index) [11]. Aytekin et al. designed a general algorithm for automatically extracting buildings from multispectral images by using spectral and spatial characteristics [12]. Jimenez et al. proposed an efficient implementation of MBI and MSI algorithms, which were specially developed for GPU [13].Jinxing et al. introduced a method based on multi-level segmentation and multi-feature fusion for building detection in remote sensing images [14]. Cui et al. proposed a method based on the Hough transform combining edge and region to extract complex buildings [15].

(B) Deep learning model: convolutional neural networks have been successfully applied to natural image categorization recently. Studies have shown that the deep learning method can effectively improve the accuracy of building extraction. Guo et al. proposed a series of convolutional neural networks that can be applied to a pixel level classification framework for township building identification [16]. Kang et al. proposed a general framework for building classification based on convolutional neural networks [17]. Makantasis et al. addressed the problem of man-made object detection from hyperspectral data through a deep learning classification framework [18]. Nogueira et al. analyzed three strategies of applying convolutional neural networks to remote sensing images [19]. The results show that fine tuning is the best training strategy of convolutional neural networks applied to remote sensing images. Yu et al. proposed a convolutional neural network remote sensing classification model based on PTL-CFS (parameter transfer learning and correlation-based feature selection), which can accelerate the convergence speed of CNN loss function [20].

Mask R-CNN was proposed after R-CNN [21], Fast R-CNN [22], and Faster R-CNN [23]. The architecture of R-CNN is divided into three parts: firstly, 2000 candidate regions are extracted by selective search; secondly, the extracted candidate regions are extracted by a multi-layer convolutional neural network; lastly, support vector machine (SVM) and linear regression model are used to classify and regress the object.

Although the R-CNN has high extraction accuracy, it is difficult to train and has lower execution time of inference. To solve this problem, Fast R-CNN and Faster R-CNN are optimized in many ways: 1. The deep convolutional neural network is used to extract the features of the original image directly. 2. The RPN (region proposal network) is used instead of the selective search to extract the candidate regions so as to reduce the training time of the model. 3. The end-to-end training is realized by using fully connected layers instead of SVM. 4. The unified feature map size of the region of interest pooling (ROI pooling) is proposed to meet the input requirements of fully connected layers. 

Mask R-CNN adds a fully convolutional network (FCN) [24] branch to Faster R-CNN to segment the object. At the same time, the ROI (region of interest) align method based on the bilinear interpolation algorithm is proposed to solve the problem of the pixel offset between the input image and the feature map caused by ROI pooling.

**Figure 2:**
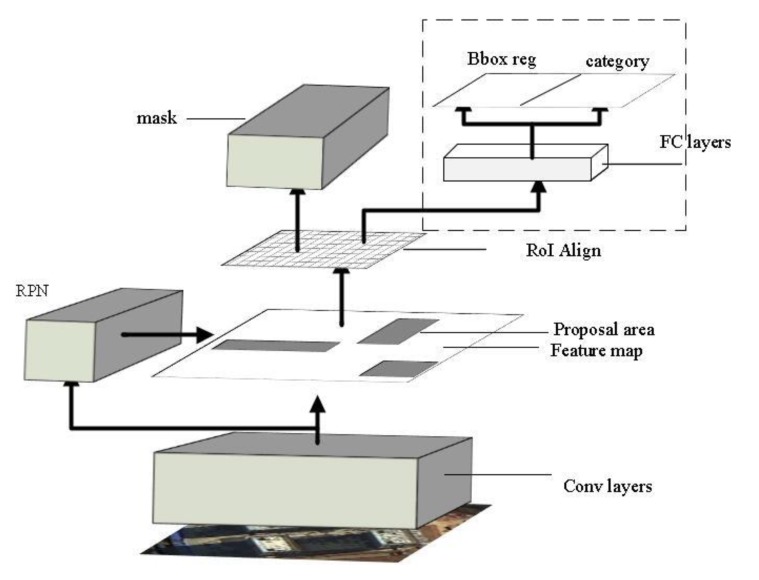
Architecture of the Mask R-CNN.

Mask R-CNN architecture is shown in Figure 2. The original image is processed by the multi-layered convolutional network to obtain hierarchical contextual image features, then the candidate region is extracted by region selection networks, and the ROI pooling of the feature map in Faster R-CNN is solved by using ROI align based on the bilinear interpolation algorithm. In addition, FCN is introduced as a branch of the model to achieve accurate segmentation of objects.

## 3. Fast and Effective Building Extraction Method

Due to the high precision of Mask R-CNN in natural image segmentation, this paper will improve the architecture of Mask R-CNN to get our framework, which is applicable to building extraction. For simplicity, we abbreviate this method to MRFS (Mask R-CNN Fusion Sobel). Our method can be summarized as follows:

(1) Image preprocessing: High resolution remote sensing images usually include panchromatic images and multispectral images. Panchromatic images have high resolution and little spectral information. Multispectral images have low resolution and rich spectral information. Both are not conducive to dense pixelwise labeling. Therefore, we enhance image information through fusing the panchromatic images and multispectral images.
(2) Constructing a network model: Our network architecture is improved based on Mask R-CNN. The feature pyramid network is utilized for feature fusion in order to improve the final detection accuracy. Compared with the Mask R-CNN model, RestNet50 (residual network) is used as the pre-training model in this paper to extract building features, and to remove the branch of boundary fitting and category judgment.
(3) Training of the network model: We adopt the cross-validation method to train our model.
(4) Detection: The trained model is used for building extraction, and the results are used as the input data of the proposed method.
(5) Combining edge features: The remote sensing image is segmented by edge features, and the building extraction results in the previous step are optimized by the results.

### 3.1. Image Preprocessing

A GF-2 remote sensing image is selected as the raw data, including the panchromatic image and multispectral image. We fuse the panchromatic images and multispectral images to get high-resolution multispectral images.

**Figure 3:**
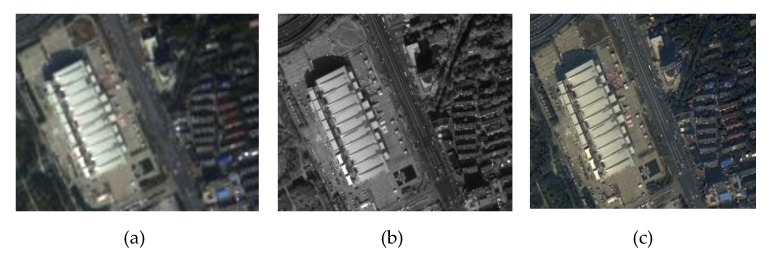
Remote sensing image. (**a**) Multispectral image; (**b**) Panchromatic image; (**c**) Fusion image based on nearest-neighbor diffusion pan-shaping algorithm.

In order to ensure that the high-resolution multispectral images can preserve the color, texture and spectral information of remote sensing images effectively, the nearest-neighbor diffusion pan-shaping algorithm is used for image fusion, and the fusion maps are shown in Figure 3.

### 3.2. Network Model

**Figure 4:**
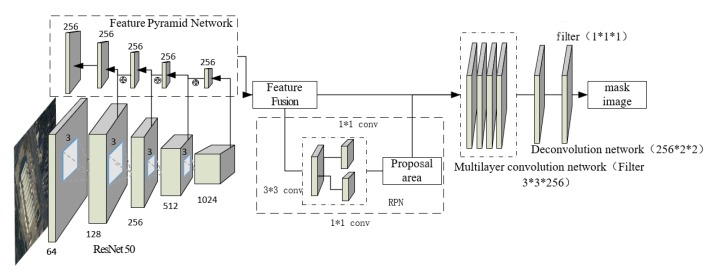
Single class extraction model of the convolution network based on Mask R-CNN.

Mask R-CNN models have made remarkable achievements in the field of image segmentation. Compared with the single-stage convolutional neural networks, Mask R-CNN has higher precision in image segmentation. The architecture of the model is shown in Figure 4. In the original image, the multi-layer convolutional neural network is used to obtain the high-dimensional feature map, and candidate regions are extracted by RPN. The corresponding position offset between the feature maps in Faster R-CNN and the original map is solved by using the pooling layer ROI align based on the bilinear interpolation algorithm. In addition, the model achieves object segmentation by fully convolutional networks as a branch.

Compared with multi-object segmentation, building extraction in remote sensing images is essentially a binary classification task. Therefore, the branch for category detection in the model is removed to optimize the training time of the model. For our improved Mask R-CNN in this paper, the cross-entropy loss function is used as follows.

#### 3.2.1. Loss function

In our network architecture, the output of the RPN recommendation area is used as the input of the fully convolutional layer, and FCN is used to complete a per-pixel classification. The cross-entropy loss function is defined as:

(1) $$\mathrm{Lx}=L_{mask}=-\frac{1}{m}\overset{m}{\underset{i=1}{\sum }}L\left(x_{i}\right)$$

(2) $$L\left(x_{i}\right)=-\frac{1}{n}\overset{n}{\underset{j=1}{\sum }}\left[y_{j}lna_{j}+\left(1-y_{j}\right)\ln\left(1-a_{j}\right)\right]$$

where L(x) denotes the loss function of the total training samples;$\;L_{mask}$ is the loss function of the mask branches; *m* is the total number of samples;$\;Lx_{i}$ is the loss value of a single sample; *n* is the number of pixels of a single sample;$\;y_{j}$ is the expected output of a single pixel; and $a_{j}$ is the output of the neural network. 

The output layer of Mask R-CNN uses the sigmoid function as the activation function, and uses the average binary value of each sample as a loss function. Cross entropy is used to train the back propagation of convolutional neural networks. Building extraction is a binary classification for a single pixel. The expected output of a single pixel can be expressed as 0 or 1. From the cross entropy function, when the expected value of a pixel is 0 or 1, and the predicted value $a_{j}$ approaches the expected value $y_{j}$, the cross entropy loss function L$\left(x_{i}\right)$ approaches 0; otherwise, L$\left(x_{i}\right)$ is close to infinity.

#### 3.2.2. Dataset Construction

We annotate different remote sensing images and use them as the experimental data of our method. The dataset includes the visual interpretation of buildings from the GF-2 remote sensing image and a building dataset from (https://www.cs.toronto.edu/~vmnih/data/). In summary, the training datasets contain 3231 images. In order to increase the sample size, the data sets are rotated at 90°, 180°, and 270°, and flipped vertically and horizontally. In order to ensure the uniform distribution of data, the data sets are randomly divided into training sets and test sets with the proportion of 7:3.

**Figure 5:**
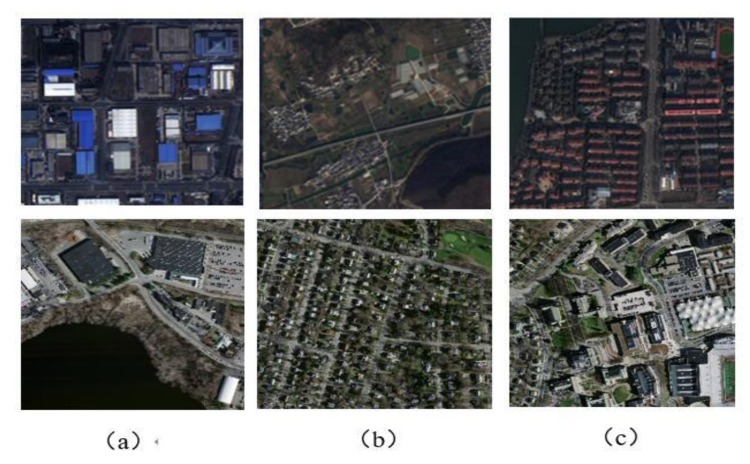
Validate dataset. (**a**) Large regular building group. (**b**) Village buildings. (**c**) Medium sized regular buildings.

In order to verify the performance of MRFS on different kinds of buildings, this paper selects three kinds of buildings according to their structure and distribution characteristics. This is shown in Figure 5.

### 3.3. Combining Artificial Edge Features

Deep convolutional networks have a high accuracy of the recognition and localization of the object, but there are certain shortcomings in the segmentation of the object, which are mainly manifested on the edge extraction and the integrity of the object. In order to solve these problems, we propose an optimization method of object extraction based on deep convolutional neural networks combined with artificial edge features.

The edge detection points of the Sobel operator are accurately located. There are fewer edge detection errors, and the operator detection edge points correspond to the actual edge points one by one. Therefore, we use the Sobel operator to obtain the gradient amplitude and gradient direction.

**Figure 6:**
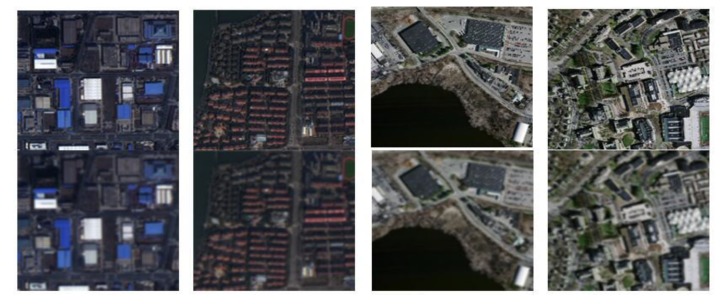
Gaussian filtering result.

In order to reduce the influence of image noise on the edge detector, a Gaussian filter is used for the smoothing operation. The Gaussian filtering results are shown in Figure 6.

For the Gaussian smoothed image, the Sobel operator is used to obtain the gradient amplitude and gradient direction. The specific formula is as follows:

(3) $$G_{X}=\left[\begin{array}{cc} \begin{array}{cc} -1 & 0 \end{array} & -1\\ \begin{array}{cc} \begin{array}{c} -2\\ -1 \end{array} & \begin{array}{c} 0\\ 0 \end{array} \end{array} & \begin{array}{c} -2\\ -1 \end{array} \end{array}\right]*\;A$$

(4) $$G_{y}=\left[\begin{array}{cc} \begin{array}{cc} +1 & +2 \end{array} & -1\\ \begin{array}{cc} \begin{array}{c} 0\\ -1 \end{array} & \begin{array}{c} 0\\ -2 \end{array} \end{array} & \begin{array}{c} 0\\ -1 \end{array} \end{array}\right]*\;A$$

where *A* denotes the original image, and $G_{X}$ and $G_{y}$ are the first derivative values in the horizontal and vertical directions. Thus, the gradient G and direction θ of the pixel can be determined.

(5) $$Μ=\sqrt{G_{x}{}^{2}+G_{y}{\;}^{2}}$$

(6) $$\theta =\arctan\left(\frac{G_{y}}{G_{x}}\right)$$

where *M* denotes the edge strength of the image; and θ the edge direction.

In order to solve the shortcomings of convolutional neural networks on building extraction, we propose optimizing the extraction results by an artificial design of edge features. 

**Figure 7:**
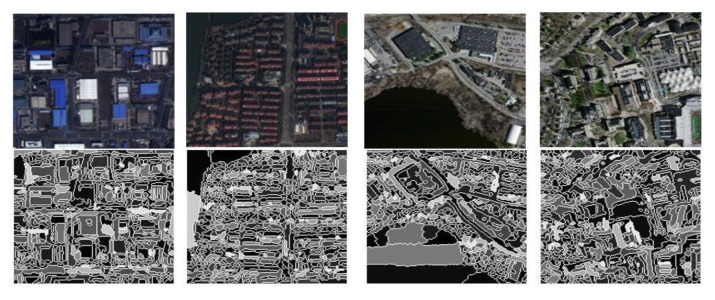
Remote sensing image after edge segmentation.

The process in detail is as follows:

- Use the Sobel operator to detect edges of remote sensing images and apply the watershed algorithm to perform label segmentation on gradient images, which is shown in Figure 7.
- The trained convolutional neural network is used to build the extraction model and get the map of the building extraction.
- Get the area of the building object in step (b) and the area of the object in the corresponding position in step (a). Establish a judgment function including the threshold value λ, as shown in formula 7. When the pixel value of the object occupied by the mask is greater than a certain threshold, the object is marked as a building object.
  (7) $$\Upsilon \left(x_{i},y_{i}\right)=sign\left(x_{i}-\lambda y_{i}\right)$$
  where γ is 0, 1 or −1 for the building mark. If γ is 1 or 0, then object *i* is marked as building, and if γ is −1, object i is marked as non-building. Here $x_{i}$ denotes the number of pixels of the mask occupying object i, $y_{i}$ is the number of pixels of object i, and λ is the threshold.

## 4. Experiments

### 4.1. Setting

All of the experiments are implemented on a single NVIDIA GeForce GTX1070 with 8 GB memory. The training time of the u-net model is 5 h. Mask R-CNN uses ResNet50 as the backbone, with a batch size of 2. We use a weight decay of 0.001 and momentum of 0.9. The training time of Mask R-CNN and the MRFS model is 2 days, and 120 K iterations are completed. We use a threshold λ = 0.6.

### 4.2. Evaluation Criteria

In this paper, the three measures, IoU, detection accuracy (pixel accuracy), and Kappa coefficients, are selected to evaluate our method. They are commonly used in remote sensing classifications. The IoU describes the degree of overlap between the predicted value and the authenticity value; the detection accuracy is used to measure the proportion of correct prediction results; the Kappa coefficient is usually used to measure the accuracy of remote sensing image classifications. Their formulae are shown in Equation (8) to Equation (10):

(8) $$\mathrm{IoU}=\frac{Area\left(P\right)\cap^{\;}Area\left(T\right)}{Area\left(P\right)\cup^{\;}Area\left(T\right)}$$

(9) $$P=\frac{TP}{TP+FP}$$

(10) $$K=\frac{p_{0}-p_{e}}{1-p_{e}}$$

In Equation (8), Area(P) is the prediction area and Area(T) is the true value area. In Equation (9), P is the detection accuracy rate, TP is the correct detection, and FP is the error detection. In Equation (10), K is the kappa coefficient, $p_{0}$ explains the proportion of correct cells, and $p_{e}$ is the proportion of misinterpretations caused by chance.

### 4.3. Analysis

#### 4.3.1. Comparison of loss function curves

**Figure 8:**
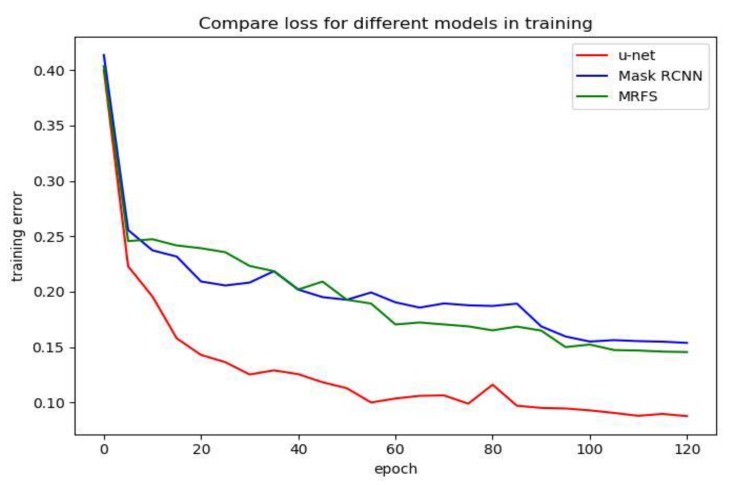
Comparison of u-net, mask R-CNN and MRFS on training error.

In order to analyze the effect of three convolution neural network models, we compared the loss function curves of u-net [7], Mask R-CNN and MRFS. As shown in Figure 8, we record the training error every five epochs and plot the loss function curve.

Compared with u-net, Mask R-CNN and the MRFS model have higher training error because the deeper network has a higher training error [10]. It can be seen from Table 1 and Figure 8 that when the training error converges to a bad local minimum, the method proposed in this paper has a better performance.

#### 4.3.2. Evaluation of different types of buildings

**Figure 9:**
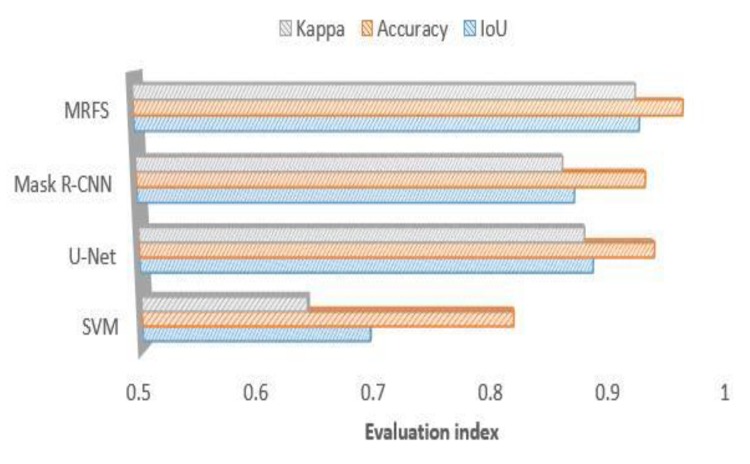
Comparison of MRFS, Mask R-CNN, SVM and KNN(k-Nearest Neighbor) on large building extractions.

**Figure 10:**
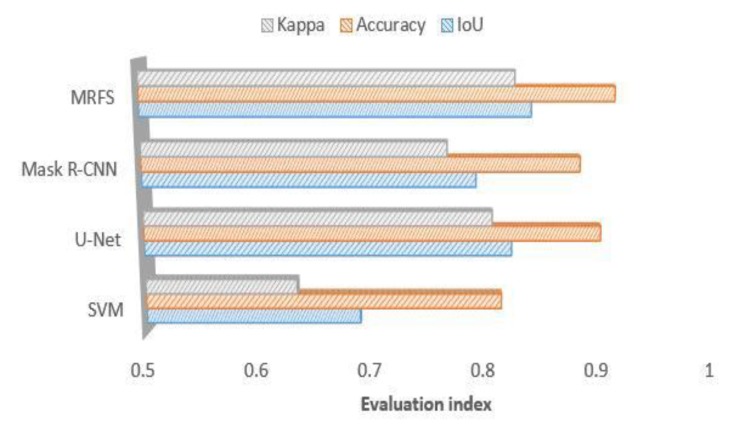
Comparison of MRFS, Mask R-CNN, u-net, SVM and KNN on the village buildings extraction.

**Figure 11:**
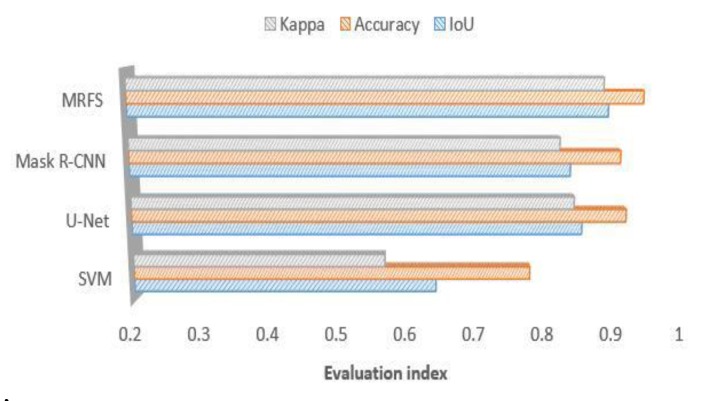
Comparison of MRFS, Mask R-CNN, SVM and KNN on medium sized area c.

We use the same dataset to train the convolutional neural networks and divide it into three parts to test according to the building characteristics. The experiments are shown in Figure 9, Figure 10 and Figure 11. This paper compares the performance of three convolution neural networks in three different regions, analyzes the shortcomings of convolution neural networks in building extraction of high-resolution remote sensing images, and verifies the effectiveness of the proposed method in this paper.

**Figure 12:**
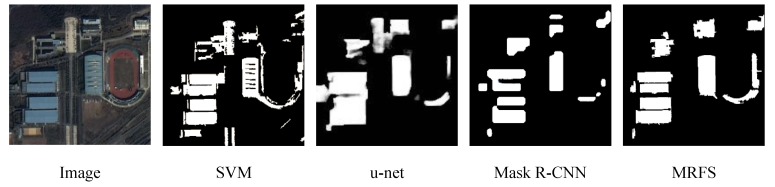
Building extraction result maps of different methods.

(1) In this paper, three evaluation indexes are used to measure the extraction results of SVM, u-net, Mask R-CNN and MRFS. Figure 9 shows the high recognition ability of the above method for large regular buildings. As shown in Figure 12, MRFS has obvious advantages in object integrity and edge extraction compared with the classical convolutional neural network algorithm.

(2) For the village buildings, Figure 10 shows that compared with the SVM, the convolutional networks model has better extraction results, which proves that the deep convolutional neural network has advantages on the extraction of complex buildings. Due to the poor edge of rural buildings, the evaluation result of MRFS is lower than that of area a.

(3) Comparing the performance of the four methods on medium sized buildings (as shown in Figure 5c), Figure 11 shows that the convolutional neural network has a high consistency with the real value of the building label. At the same time, it shows that the artificial feature can optimize the result of the convolution neural network.

#### 4.3.3. Comparison of Single building extraction

In order to further analyze the efficiency of these four methods in remote sensing image building extraction, Figure 12 shows the building extraction map of the four methods, where the black and the white represent background and building respectively. SVM is conducive to the overall recognition of buildings, but it cannot effectively distinguish between the cement floors and buildings, and there are a lot of false extractions. Mask R-CNN and u-net have achieved high accuracy in building recognition and building locating, However, compared with visual interpretation, they are still insufficient on edge extraction and object integrity.

The main error sources of classical convolution neural networks are as follows: firstly, a convolutional neural network has a high requirement on hardware equipment. The batch size in this paper is 2. In convolutional neural networks, large batches usually make the network converge faster, but due to the limitation of memory resources, large batches may lead to insufficient memory or program kernel crash. Secondly, in the convolution neural network, the pooling layer is used to reduce the model parameters, and the deconvolution operation will also affect the integrity of object extraction to a certain extent. In summary, the difference between the proposed method and the classical convolutional neural network in building recognition is mainly focused on the optimization of building edges, which can solve the incompleteness of the building extraction.

**Figure 13:**
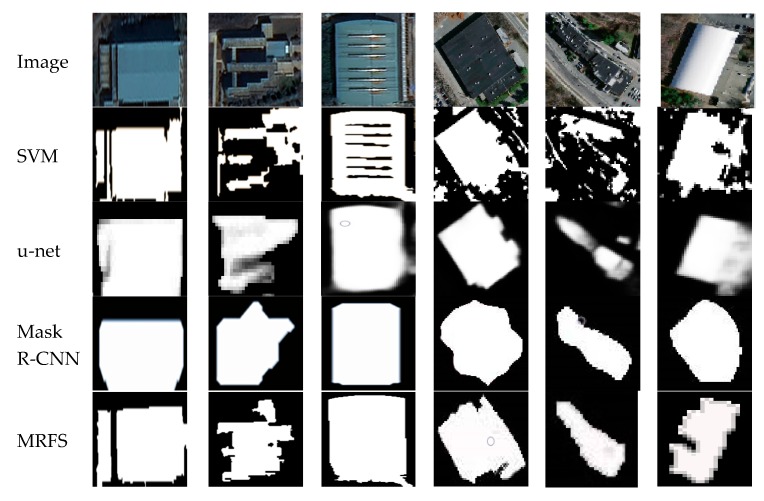
Comparison of single building detection maps.

Figure 12 shows that the convolutional neural network performs poorly in edge extraction and the integrity of the object but the MRFS can deal with such problems more effectively. Figure 13 shows the extracted results of the single building objects after enlargement. Compared with convolution neural networks, the support vector machine has better edge segmentation, but misses some areas of complex buildings. For Mask R-CNN, u-net and MRFS proposed in this paper, the convolutional neural network has better efficiency to locate buildings in complex areas. 

Table 1 shows that due to the complex structure and various materials of remote sensing image buildings, object-oriented building extraction methods are prone to large-scale error recognition, and convolutional neural networks solve this problem well. The MRFS method we proposed in this paper solves the problems of edge extraction and the integrity of the object. Therefore, compared with the Mask R-CNN and other methods, our method has better efficiency on different building extractions.

#### 4.3.4. Edge feature fusion parameter λ

**Figure 14:**
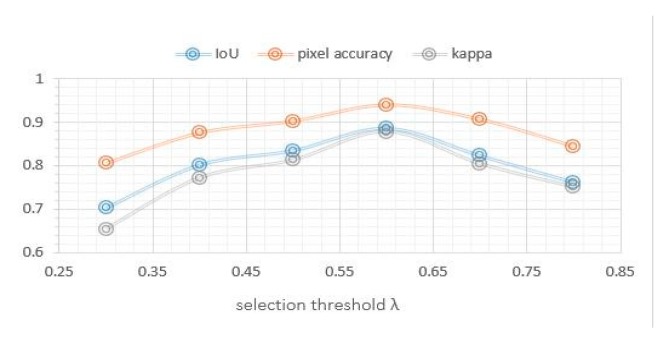
Effect of threshold parameter λ on accuracy.

In order to analyze the influence of the parameters of the MRFS, this paper carries out a number of experiments by changing the object selection threshold parameter λ. The values of λ are set as 0.3, 0.4, 0.5, 0.6, 0.7 and 0.8, respectively. Figure 14 shows the changes of IoU, detection accuracy and kappa with the threshold parameter λ. In this experiment, λ is in the range of [0.4,0.6], and the change of each index is relatively small.

## 5. Conclusions

In this paper, the design of deep convolutional neural networks in semantic segmentation is applied to extract building from high-resolution remote sensing images. Based on the characteristics of the deep convolutional network, building recognition and high-precision extraction in high-resolution remote sensing images were realized. Concerning the problems of poor edge recognition and incomplete extraction of the convolutional networks on the building extraction from remote sensing images, an optimization method combining edge features was proposed to improve the efficiency of the network model on building extraction. Experiments on 3231 images and 20,000 building objects were carried out to verify the effectiveness of the method we proposed in this paper. Compared with the classical convolutional network models, the accuracy rate and integrity of building extraction were improved. In the future, the relationship between the selection of the threshold parameter λ and the Mask R-CNN training results will be analyzed in detail, and the method will be improved by automatically getting the model parameters.

## Figures and Tables

**Table 1:** Quantitative evaluation of different methods on building extraction.

Test Area	IoU				Pixel Accuracy				Kappa			
	SVM	u-net	Mask R-CNN	MRFS	SVM	u-net	Mask R-CNN	MRFS	SVM	u-net	Mask R-CNN	MRFS
Area a	0.697	0.890	0.871	0.925	0.822	0.941	0.931	0.961	0.643	0.881	0.861	0.921
Area b	0.691	0.826	0.794	0.841	0.818	0.905	0.885	0.914	0.634	0.808	0.768	0.827
Area c	0.647	0.861	0.841	0.894	0.786	0.925	0.914	0.943	0.571	0.848	0.825	0.886
mean	0.679	0.859	0.836	0.887	0.808	0.924	0.910	0.940	0.616	0.846	0.818	0.878
